# Supplementary material for: Modeling glioblastoma heterogeneity as a dynamic network of cell states
Source: Mol Syst Biol. 2021 Sep 16;17(9):e10105. doi: 10.15252/msb.202010105 (PMC8444284; doi:10.15252/msb.202010105)
Supplement: Supplementary file 6 — Source Data for Figure 5 [file MSB-17-e10105-s004.zip › Figure5A_sourcedata/GSEA_3071/nosigdb_stateVI.GseaPreranked.1622795169056/SUVA_AC_PMID_31327527.html]

Details for gene set SUVA\_AC\_PMID\_31327527[GSEA]

|  || Dataset | state63071 |
| Phenotype | NoPhenotypeAvailable |
| Upregulated in class | na\_neg |
| GeneSet | SUVA\_AC\_PMID\_31327527 |
| Enrichment Score (ES) | -0.7797827 |
| Normalized Enrichment Score (NES) | -2.7586257 |
| Nominal p-value | 0.0 |
| FDR q-value | 0.0 |
| FWER p-Value | 0.0 |
Table: GSEA Results Summary

  

Fig 1: Enrichment plot: SUVA\_AC\_PMID\_31327527      
 Profile of the Running ES Score & Positions of GeneSet Members on the Rank Ordered List

  

| PROBE | GENE SYMBOL | GENE\_TITLE | RANK IN GENE LIST | RANK METRIC SCORE | RUNNING ES | CORE ENRICHMENT || 1 | F3 |  |  | 26 | 0.988 | 0.0270 | No |
| 2 | HOPX |  |  | 89 | 0.761 | 0.0362 | No |
| 3 | GATM |  |  | 2082 | -0.292 | -0.5042 | No |
| 4 | RAB31 |  |  | 2806 | -0.459 | -0.6882 | No |
| 5 | TSPAN7 |  |  | 2848 | -0.475 | -0.6831 | No |
| 6 | PON2 |  |  | 2884 | -0.490 | -0.6758 | No |
| 7 | HEPN1 |  |  | 2941 | -0.510 | -0.6736 | No |
| 8 | MLC1 |  |  | 3326 | -0.793 | -0.7523 | Yes |
| 9 | DBI |  |  | 3349 | -0.812 | -0.7303 | Yes |
| 10 | SPARC |  |  | 3356 | -0.822 | -0.7035 | Yes |
| 11 | PRCP |  |  | 3417 | -0.927 | -0.6880 | Yes |
| 12 | SLC1A3 |  |  | 3458 | -1.025 | -0.6636 | Yes |
| 13 | PCDHGC3 |  |  | 3472 | -1.051 | -0.6308 | Yes |
| 14 | TTYH1 |  |  | 3481 | -1.067 | -0.5961 | Yes |
| 15 | CLU |  |  | 3601 | -1.801 | -0.5666 | Yes |
| 16 | GPM6B |  |  | 3616 | -2.257 | -0.4924 | Yes |
| 17 | GFAP |  |  | 3619 | -2.337 | -0.4120 | Yes |
| 18 | PMP2 |  |  | 3622 | -2.497 | -0.3261 | Yes |
| 19 | S100B |  |  | 3624 | -2.502 | -0.2398 | Yes |
| 20 | FABP7 |  |  | 3629 | -2.788 | -0.1444 | Yes |
| 21 | PTPRZ1 |  |  | 3637 | -4.237 | 0.0003 | Yes |
Table: GSEA details [plain text format]

  

Fig 2: SUVA\_AC\_PMID\_31327527: Random ES distribution      
 Gene set null distribution of ES for **SUVA\_AC\_PMID\_31327527**
